# Identification and validation of senescence-related genes in polycystic ovary syndrome

**DOI:** 10.1186/s13048-023-01338-4

**Published:** 2024-01-06

**Authors:** Danni Jiang, Yang Xu, Lin Yang, Pengfei Li, Xiaojuan Han, Qianni Li, Yang Yang, Lan Chao

**Affiliations:** 1 Center for Reproductive Medicine, Department of Obstetrics and Gynecology, Qilu Hospital, Shandong University, Jinan, China; 2 Department of Reproductive Medicine, Linyi People’s Hospital, Shandong University, Linyi, China

**Keywords:** Polycystic ovary syndrome, Aging, Gene, Bioinformatics

## Abstract

**Background:**

Polycystic ovary syndrome (PCOS) is an exceedingly intractable issue affecting female endocrine and reproductive health. However, the etiology and intricate pathological mechanisms of PCOS remain unclear. Nowadays, aging was found to share multiple common pathological mechanisms with PCOS, which causes probing into the pathogenesis of PCOS from senescence. However, no bioinformatics analyses have specifically focused on connection between PCOS and ovarian aging.

**Methods:**

Differentially expressed aging-related genes in PCOS were identified and then analyzed using function enrichment method. Hub genes were determined based on multiple algorithms, and expression validation of hub genes was performed in both datasets and experiments (human granulosa-like tumor cell line, KGN; human Granulosa Cell, hGCs). Finally, a transcription factor-miRNA-gene network of hub genes was constructed.

**Results:**

Here, we identified 73 aging-related differential expression genes (ARDEGs) by intersecting DEGs in PCOS and senescence-related gene set. Furthermore, we performed biological functions and potential pathways of ARDEGs and potential hub genes were also screened by multiple algorithms. From the perspective of immune dysfunction, we analyzed the correlation between PCOS and immune cells. Finally, TF-miRNA-gene networks were constructed. Finally, TF-miRNA-gene networks were constructed.

**Conclusions:**

Our work aimed to elucidate the relation between PCOS and cellular senescence based on bioinformatics strategy, deepening the understanding of mechanisms and to seek for novel therapy strategies for improving reproductive lifespan and female health. Exploring the potential molecular mechanism of cell aging in PCOS is expected to bring a new breakthrough for PCOS diagnosis and therapy strategies. And this, might deepen our understanding about intricate mechanisms of ovarian aging.

**Supplementary Information:**

The online version contains supplementary material available at 10.1186/s13048-023-01338-4.

## Background

Polycystic ovary syndrome (PCOS), a common female endocrine abnormality, is characterized by hyperandrogenism, ovulation dysfunction and ovarian polycystic morphology [1]. It’s not only the leading cause for female infertility but also contributes to a higher risk of obesity, hypertension, insulin resistance, type 2 diabetes, cardiovascular disease, and endometrial carcinoma [2]. However, the pathological mechanism of development and progression of polycystic ovary syndrome is still unclear.

Cellular senescence, also known as senescence-associated secretory phenotype (SASP), is a state of standing cell cycle arrest manifested by significant secretion of various bioactive molecules such as reactive oxygen species, pro-inflammatory cytokines, and chemokines [3]. Cellular senescence plays a central role in aging-related organ malfunction and it can be induced by aging in a variety of cells [3].

The primordial follicular pool, responsible for the women fertility and reproductive lifespan, has been established during the embryonic life and depleted with age in most of mammalians. Of note, granulosa cells (GCs) play an important part in maintaining the follicle pool [4]. Besides, GCs surround the oocyte, produce growth factors and sex steroids, and overall contribute to oocyte development [5]. With the process of GCs aging, meiosis progress is disturbed and led to oocyte dysfunction. And this, eventually causes the faliure in maturation and full developmental potential acquisition of oocyte [6]. In recent decades, with the progress of research on aging and PCOS, studies had revealed that PCOS and cellular senescence share some common pathogenesis, such as oxidative stress, inflammatory state, immune dysfunction, and so on [1–3, 7]. However, the pathophysiologies common to both between cellular senescence and PCOS is still unclear.

Gene expression analysis has become increasingly significant in biological research. Today, biomedicine is greatly facilitated by revolutionary developments in microarray technology and bioinformatics, which allow us to explore disease-related alterations in transcriptional and related biological pathways. Several published studies have used gene expression datasets obtained from the Gene Expression Omnibus (GEO) database to illuminate the biologic mechanisms underlying the pathogenesis of PCOS [2, 8, 9]. The results of those bioinformatic analyses provide ponderable hints to researchers for comprehending the pathogenesis of PCOS from different perspectives. However, no studies focus on the pathophysiology between aging and PCOS using bioinformatics analysis, and much potentially valuable information still waits to be revealed.

This study aims to analyze the PCOS-associated datasets from an angle of the aging-related genes (ARGs). The Aging-related differentially expression genes (DEGs, ARDEGs) were obtained by intersecting of DEGs and 1230 aging and senescence-associated genes. The potential biological functions and pathways of ARDEGs were analyzed by Gene Ontology (GO), Kyoto Encyclopedia of Genes and Genomes (KEGG), Gene Set Enrichment Analysis (GSEA). Then, Weighted correlation network analysis (WGCNA) and Support vector machine-recursive feature elimination (SVM-RFE) algorithms were performed to identify potential hub genes involved in PCOS development. The expression level and diagnostic activity were validated using internal, external datasets, and receiver operating characteristic (ROC) curve. Additionally, the relevance between hub ARDEGs and immune infiltration within PCOS was explored. Finally, we conducted Transcription factor-microRNA (TF-miRNA) network of hub genes to explore the regulatory relationship between genes.

## Results

### Data processing and identification of DEGs

**Fig. 1:**
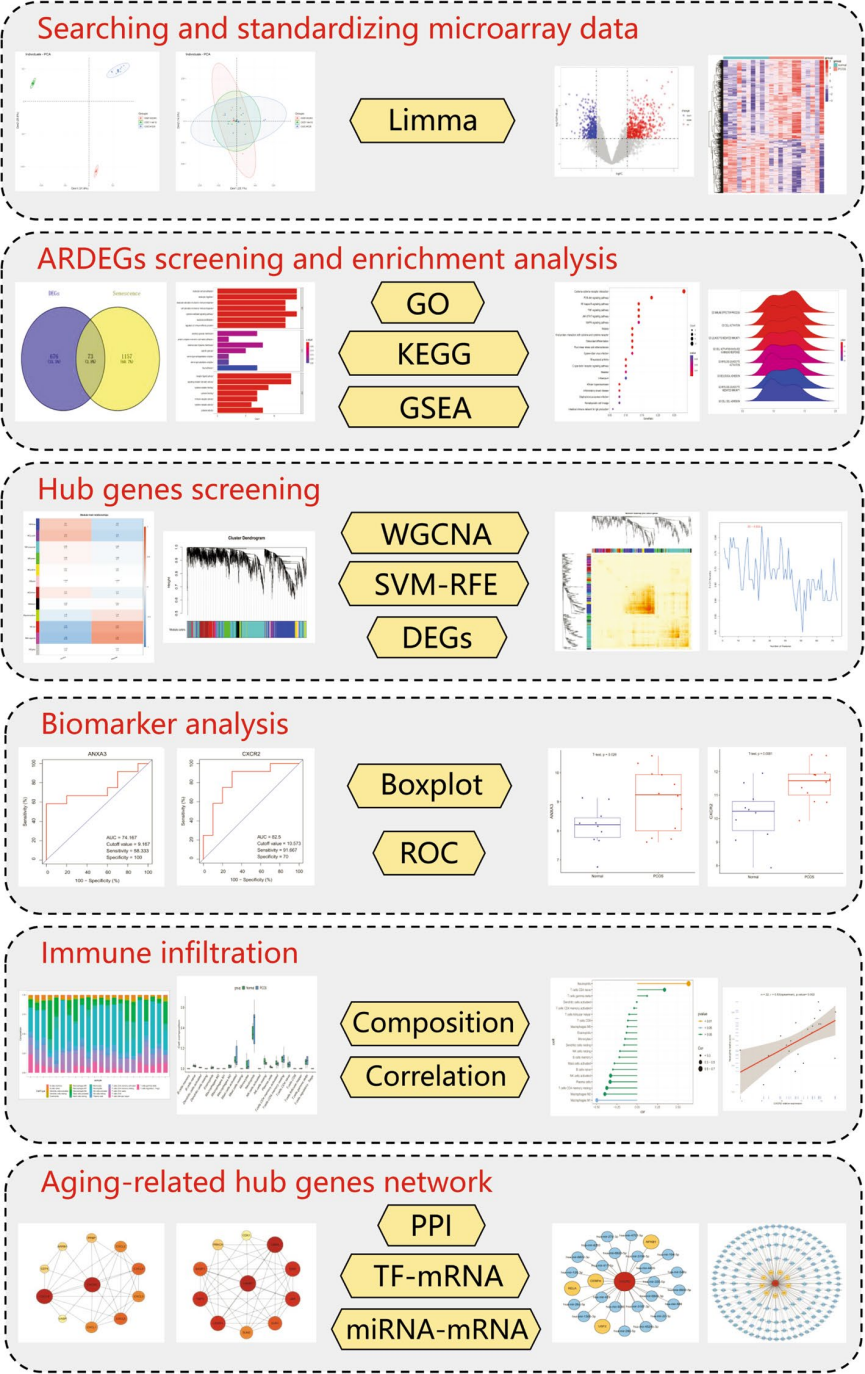
The flow diagram of the study

**Fig. 2:**
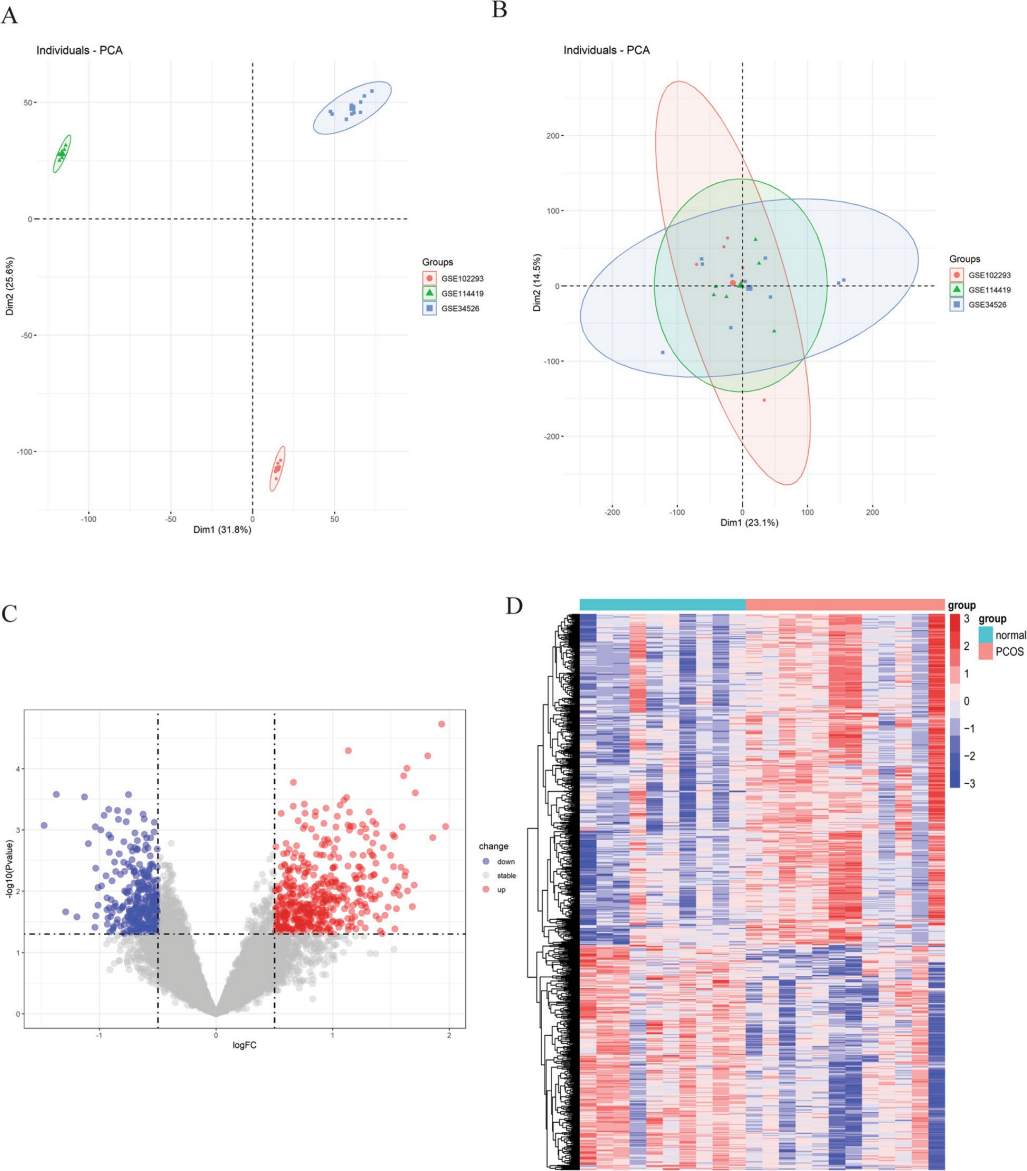
Screening of DEGs. **A** Principal component analysis. **B** Volcano plot showing the DEGs among PCOS and unaffected controls. **C** Heatmap showing DEGs expression patterns

The flow diagram of the study is presented in Fig. 1. And the details of included datasets are listed in Table 1. We merged and normalized the expression matrix, then displayed it in principal component analysis (PCA) plots before and after the standardized processing (Fig. 2A,B). Then 446 up-regulated and 303 down-regulated genes were identified and displayed in volcano and heatmap plots (Fig. 2C,D).

**Table 1:** Lists of datasets in this study

Dataset	Organism	Sample	Data type	Platform	Control	PCOS	Description
GSE34526	Homo sapiens	Granulosa cells	mRNA	GPL570	3	7	Training group
GSE102293	Homo sapiens	Granulosa cells	mRNA	GPL570	4	2	Training group
GSE114419	Homo sapiens	Granulosa cells	mRNA	GPL17568	3	3	Training group
GSE106724	Homo sapiens	Granulosa cells	mRNA/lncRNA	GPL21096	4	8	Testing group

### Identification of ARDEGs and enrichment analysis

**Fig. 3:**
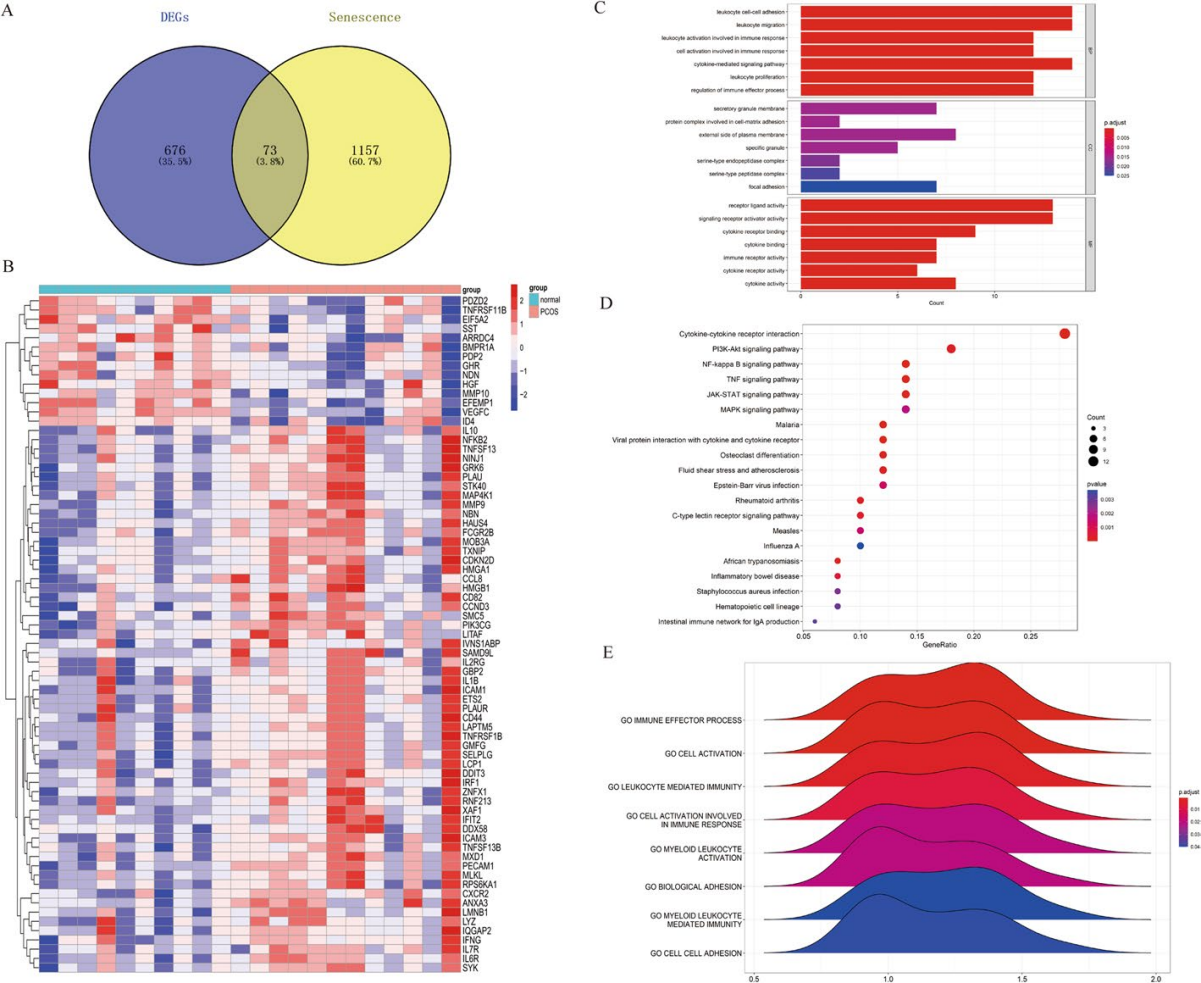
ARDEGs identification and enrichment analysis. **A** Screening ARDEGs with a venn diagram. **B** Heatmap showing ARDEGs expression. **C** GO analyses of ARDEGs. **D** KEGG pathways of ARDEGs. **E** GSEA showing the pathways that closely related to PCOS group

Totally, we obtained 1230 aging and senescence-associated genes after deleting duplicated genes; they are all listed in Supplementary Table S2. We intersected the DEGs and ARGs to screen out the ARDEGs. A total of 73 overlapped genes were revealed by venn plot (Fig. 3A), the genes were also visualized with a heatmap (Fig. 3B).

The BP results showed that the leukocyte cell–cell adhesion, leukocyte migration, leukocyte activation, cell activation involved in immune response, and cytokine-mediated signaling pathway were enriched (Fig. 3C). Besides, secretory granule membrane, protein complex involved in cell–matrix adhesion, external side of plasma membrane, specific granule, side, function including receptor ligand activity, signaling receptor activator activity, cytokine receptor binding, cytokine binding, and immune receptor activity were abundant in this category. The pathway analysis revealed that the cytokine-cytokine receptor interaction, Malaria, nuclear factor kappa-B (NF-κB) signal pathway, tumor necrosis factor (TNF) signal pathway, and Viral protein interaction with cytokine and cytokine receptor were the top 5 signal pathways enriched in KEGG terms (Fig. 3D). Moreover, we used GSEA to appraise the functional difference of ARDEGs between the PCOS and control samples. The GSEA result showed that ARDEGs were primarily involved in immune effector process, cell activation, leukocyte mediated immunity, cell activation involved in immune response, and myeloid leukocyte activation (Fig. 3E).

### Co-expression network and hub aging-related DEGs

**Fig. 4:**
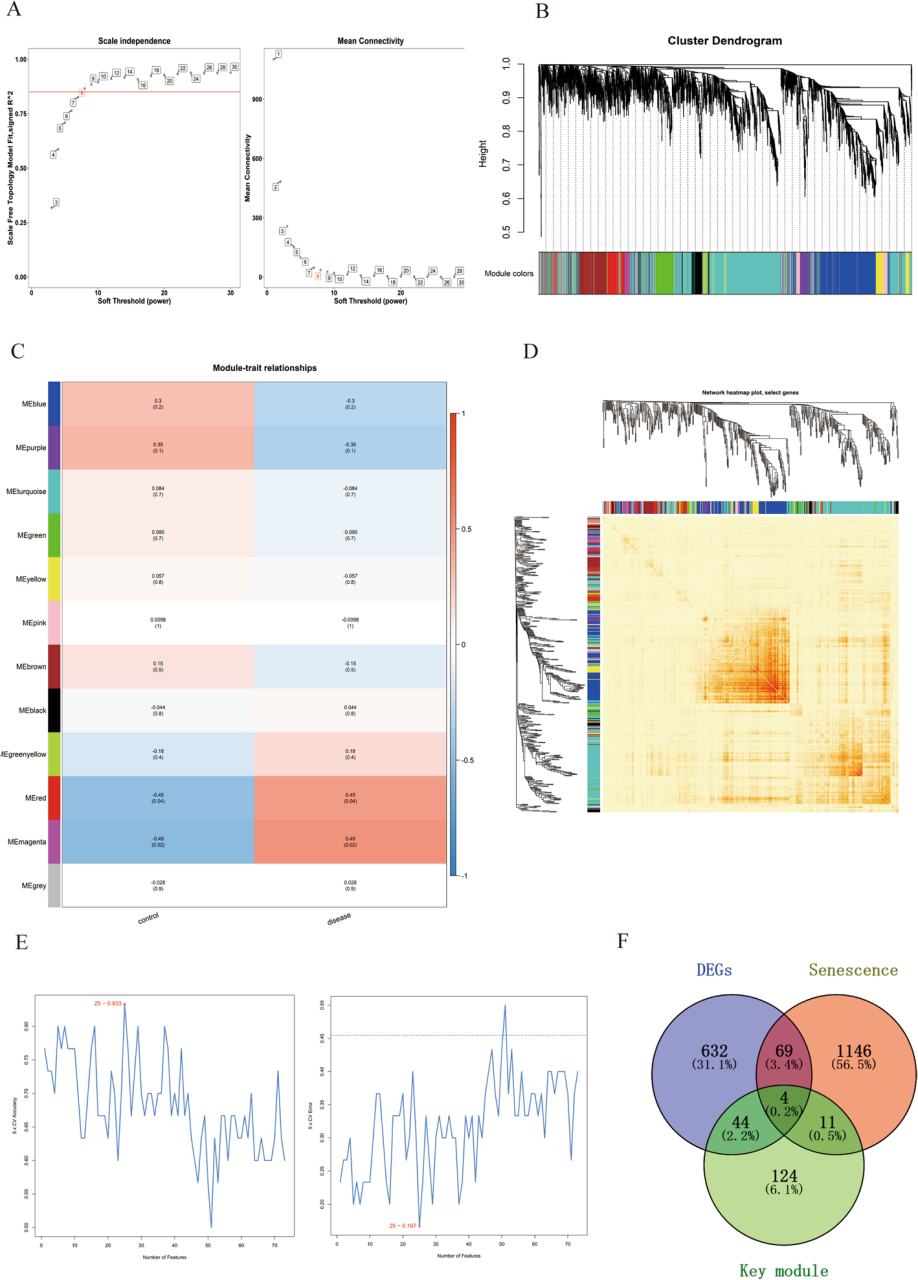
Identification of age-related hub genes in PCOS. **A** Analysis of the scale-free fit index and the mean connectivity. **B** Clustering dendrogram of DEGs related to PCOS. **C** Correlation of different modules with samples. **D** Correlation heatmap between genes. **E,F** Screening hub genes based on SVM-RFE algorithm. **G** Showing genes overlapped in DEGs, key modules, and ARGs with Venn diagram

To identify aging-related hub genes of PCOS, two methods were applied. In the WGCNA algorithm, a soft-thresholding power of 8 was used to obtain approximate scale-free topology for the network (Fig. 4A). The genes with top 5000 variance were clustered into twelve modules (Fig. 4B), two modules (magent and purple) with a total of 183 genes were closely related to the disease. The clustering dendrogram showing the relation between eigengene and clinical characteristics can be seen in Fig. 4C. Correlation heatmap between genes was plotted based on topological overlap matrix (Fig. 4D).

In addition, the 73 ARDEGs were screened as crucial genes using the SVM-RFE algorithm. In total, 25 genes were identified as key candidate biomarkers including VEGFC, CXCR2, PECAM1, SMC5, DDIT3 and etc. (Fig. 4E,F). Besides, the genes overlapped in DEGs, genes from WGCNA key modules, and ARGs were obtained (Fig. 4G). The genes in WGCNA key modules and SVM-RFE algorithm were shown in Supplementary Table S3. Through the above two algorithms, we finally obtained the 4 key genes, namely ANXA3, CXCR2, IQGAP2 and LMNB1. The comprehensive information of the four molecules were shown in Table 2.

**Table 2:** Localization and function of Aging-related PCOS hub genes

Gene	Description	Subcellular location	Expression in granulosa cells	Function
ANXA3	Annexin A3	Plasma membrane	Low	This gene encodes a member of the annexin family. Members of this calcium-dependent phospholipid-binding protein family play a role in the regulation of cellular growth and in signal transduction pathways. This protein functions in the inhibition of phopholipase A2 and cleavage of inositol 12-cyclic phosphate to form inositol 1-phosphate
CXCR2	C-X-C motif chemokine receptor	Plasma membrane In addition localized to the Nucleoplasm Microtubules Mitotic spindle	High	This protein is a receptor for interleukin 8 (IL8). It binds to IL8 with high affinity and transduces the signal through a G-protein activated second messenger system. This receptor also binds to chemokine (C-X-C motif) ligand 1 (CXCL1/MGSA) a protein with melanoma growth stimulating activity and has been shown to be a major component required for serum-dependent melanoma cell growth. This receptor mediates neutrophil migration to sites of inflammation. The angiogenic effects of IL8 in intestinal microvascular endothelial cells are found to be mediated by this receptor
IQGAP2	IQ motif containing GTPase activating protein 2	Vesicles Plasma membrane	Low	This gene encodes a member of the IQGAP family. The encoded protein contains three IQ domains one calponin homology domain one Ras-GAP domain and one WW domain. This protein interacts with components of the cytoskeleton with cell adhesion molecules and with several signaling molecules to regulate cell morphology and motility. It also acts as a tumor suppressor and has been found to play a role in regulating innate antiviral responses
LMNB1	Lamin B1	Nuclear membrane	High	This gene encodes one of the two B-type lamin proteins and is a component of the nuclear lamina. A duplication of this gene is associated with autosomal dominant adult-onset leukodystrophy (ADLD)

### Predicted performance of hub biomarkers

**Fig. 5:**
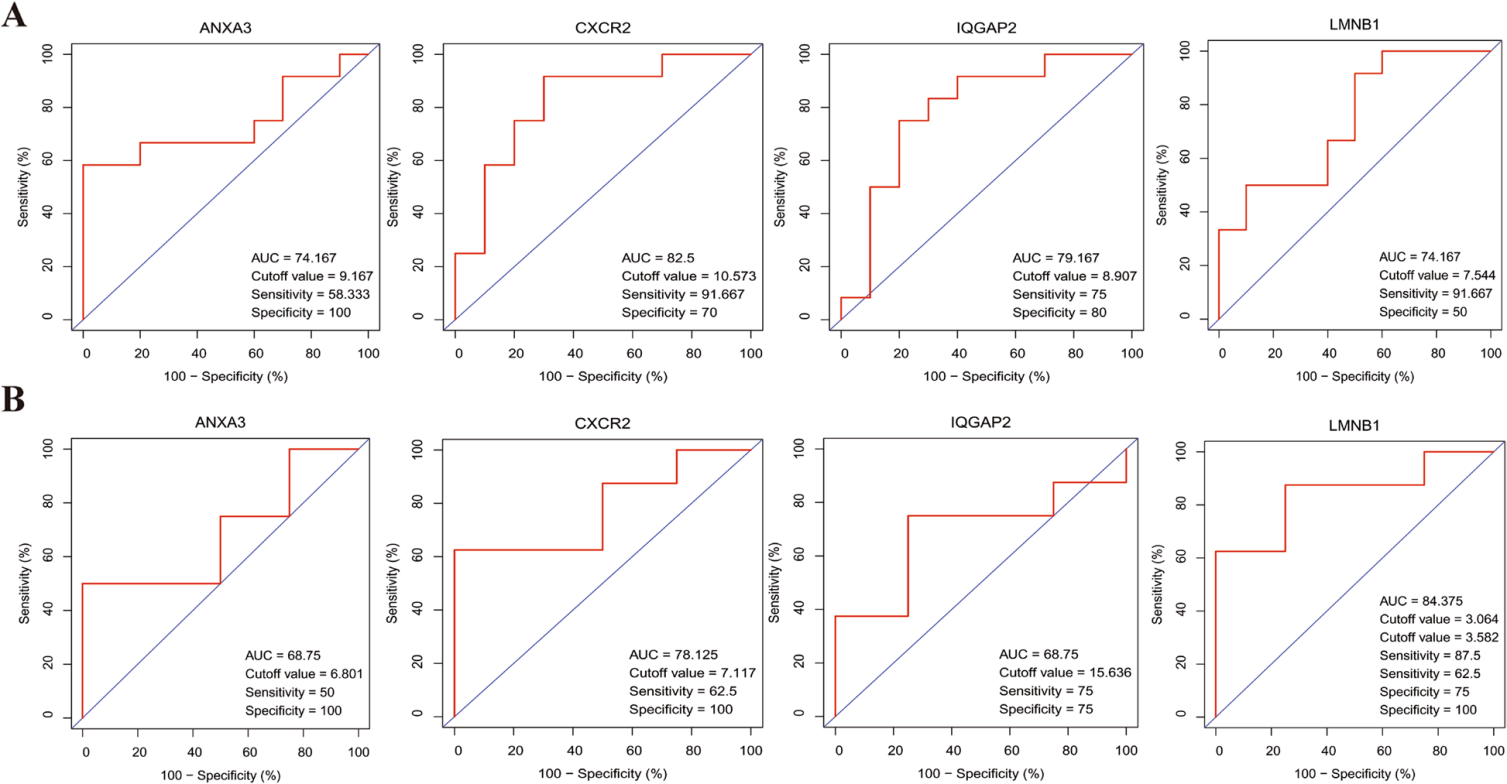
Diagnostic value of ARDEGs. **A** The ROC curves of four hub ARDEGs in the train group. **B** The ROC curves of four hub ARDEGs in the test group. ARDEGs, Aging-related differential expression genes

**Fig. 6:**
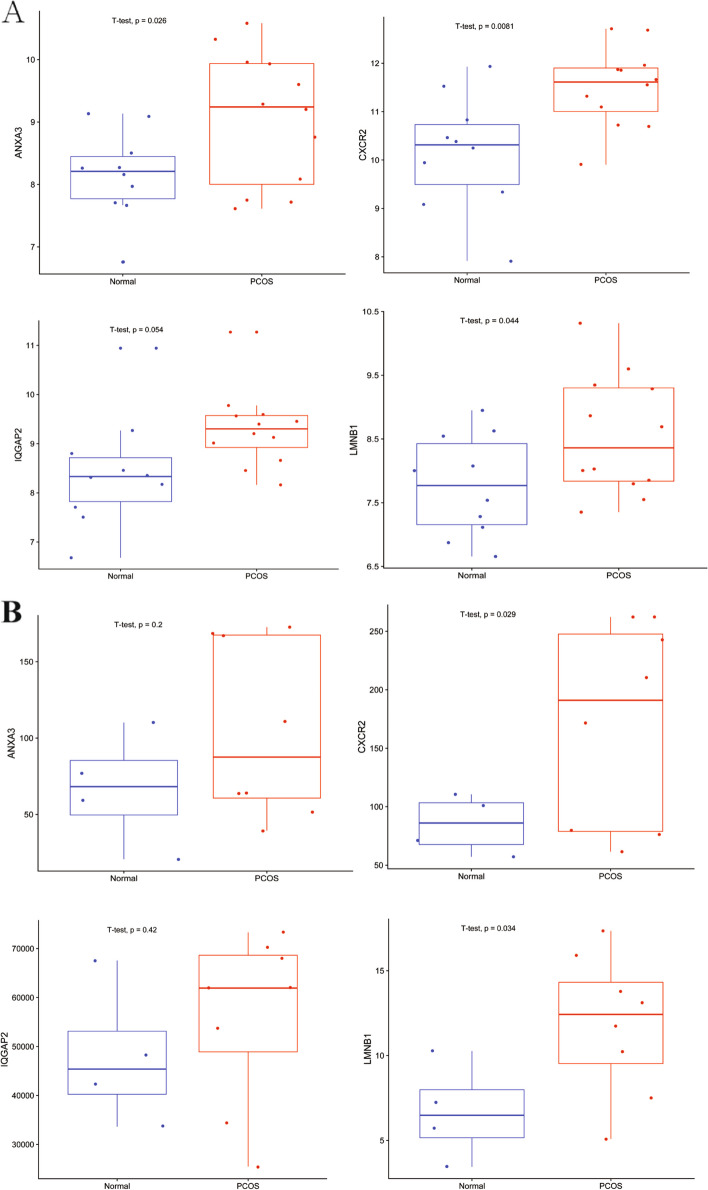
Expression level of hub genes. **A** The expression of four hub ARDEGs in the train group. **B** The expression of four hub ARDEGs in the test group

To further identify the hub genes from the four candidate biomarkers, the ROC curve was performed for training dataset. The results showed that the all of the four biomarkers had an AUC of > 0.7 for predicting PCOS (Fig. 5A). Moreover, one extra dataset (GSE106724) was used to verify the above result as a testing group. The ROC analyses indicated that only CXCR2 and LMNB1 had an AUC of 0.78 and 0.84, respectively (Fig. 5B). We also validated the expression of four genes, differential expression of these genes, namely ANXA3,CXCR2, and LMNB1, was verified in the training group (Fig. 6A). Furthermore, only two genes (CXCR2 and LMNB1) showed the difference in gene expression in the testing dataset (Fig. 6B). As above-mentioned, ANXA3 and IQGAP2 showed unsatisfactory result for both diagnostic performance and expression difference. Therefore, we obtained two hub biomarkers, namely CXCR2 and LMNB1.

### Validation of hub biomarkers

**Fig. 7:**
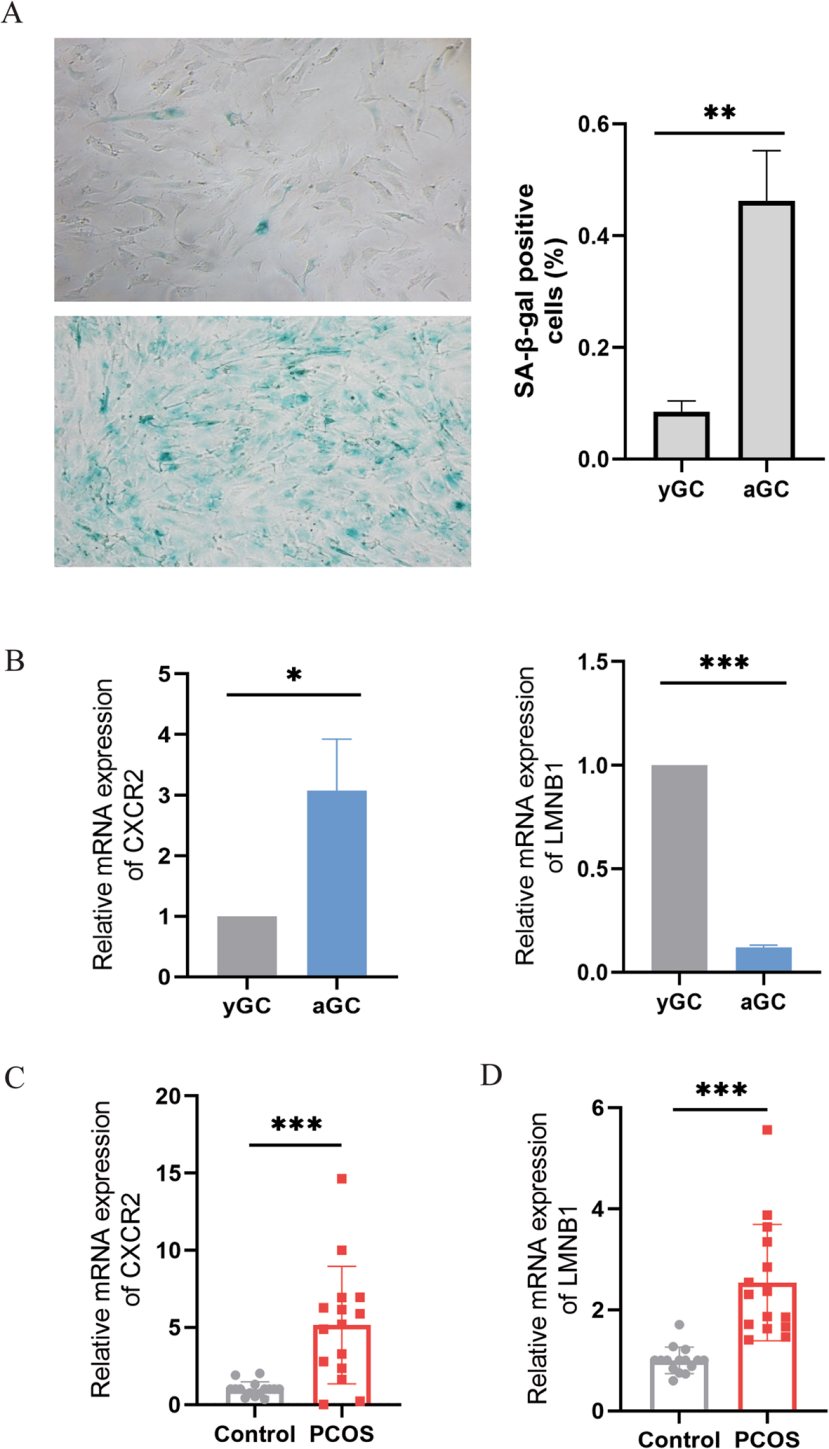
Validation of hub biomarkers. **A** Representative SA-β-gal staining for GCs from control and 5 μM ETO treatments. **B** Relative expression of CXCR2 and LMNB1 in aGCs by RT-qPCR. **C** Relative expression of CXCR2 in PCOS. **D** Relative expression of LMNB1 in PCOS

To verify the result of the bioinformatic analysis, the etoposide-induced aged granulosa cells (aGCs) model was established. The SA-β-gal assay validated the senescence phenotype (Fig. 7A). Using PCR analysis, we identified that CXCR2 was significantly upregulated and LMNB1 was significantly downregulated in aGCs when compared with young GCs (Fig. 7B). The baseline information for recruited women in this study was shown in Table 3.The above two genes were found up-regulated in PCOS patients when compared to the control group (Fig. 7C, D).

**Table 3:** Baseline characteristics of study participants

Basic parameters	Control (*n* = 15)	PCOS (*n* = 15)	*P* value
Age, year	29.8 (25,33)	28.3 (20,36)	0.3959
BMI, kg/m^2^	23.5 ± 3.4	26.3 ± 5.0	0.1117
E2, pg/ml	47.7 ± 24	45 ± 16	0.7170
T, ng/ml	0.4 ± 0.3	0.7 ± 0.6	0.3925
FSH, mIU/ml	5.6 ± 1.4	5.3 ± 1.2	0.4461
LH, mIU/ml	5.9 ± 3.0	8.1 ± 3.5	0.0930

*Abbreviation*: *BMI* body mass index, *FSH* follicle-stimulating hormone, *LH* luteinizing hormone. Data with normal distribution are shown a mean ± standard deviation (SD)

### Immune cell infiltration analysis

**Fig. 8:**
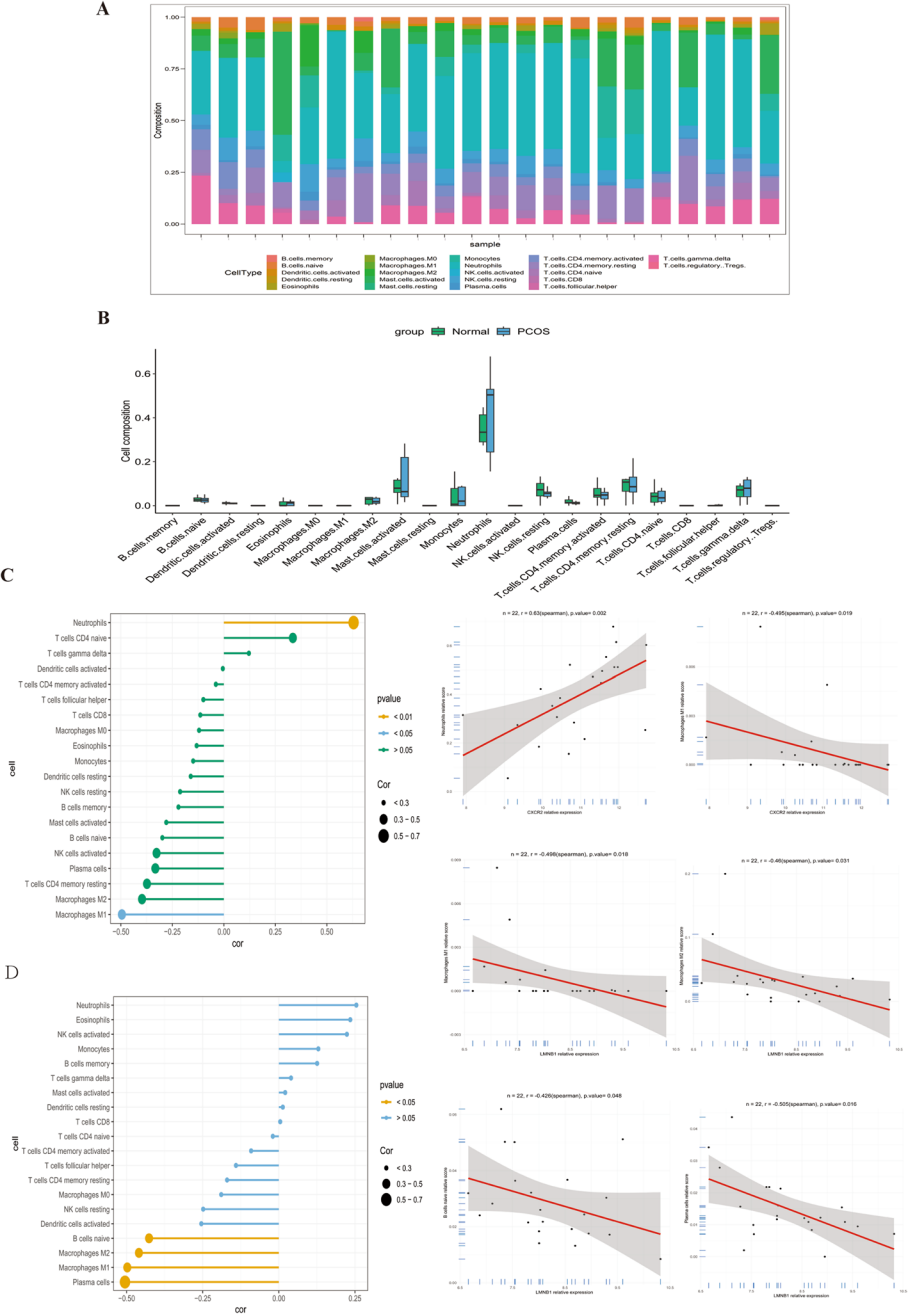
Immune cell infiltration of PCOS and its correlation with the aging-related hub genes. **A** Immune cell composition between two groups. **B** Violin plot to visualize the differentially infiltrated immune cells among two groups. **C** Correlation between CXCR2 and infiltrating immune cells. **D** Correlation between LMNB1 and infiltrating immune cells

The immune cells surrounding GCs were predicted. Compared to other immune cells, neutrophils and mast cells activated dominated in both PCOS and control groups (Fig. 8A). However, we detected no significant difference in immune cell infiltration among the groups (Fig. 8B). Moreover, we conducted correlation analysis to evaluate the correlation between hub genes and immune cells. The result revealed that the hub gene CXCR2 showed positive correlation with neutrophils (*R* = 0.630, *P* = 0.002), but negatively correlated with macrophage M1 cells (*R* =  − 0.495, *P* = 0.019; Fig. 8C). Besides, we found that LMNB1 was negatively related with plasma cells (*R* =  − 0.505, *P* = 0.016), macrophages M1 (*R* =  − 0.498, *P* = 0.018), macrophages M2 (*R* =  − 0.46, *P* = 0.031), and B cells naive (*R* =  − 0.426, *P* = 0.048; Fig. 8D).

### PPI network identification and construction of coregulatory network

**Fig. 9:**
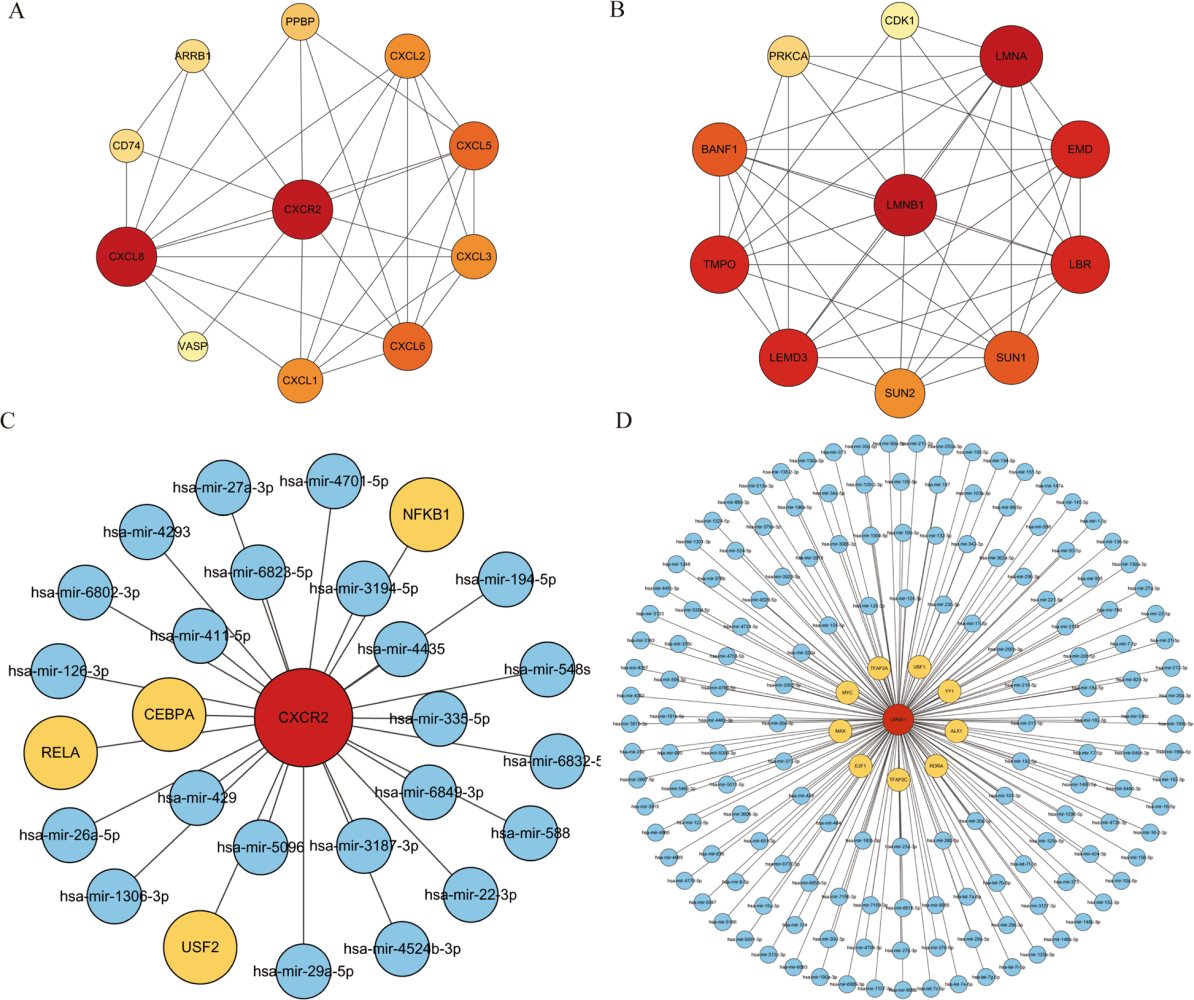
PPI analysis and regulatory network of hub genes. **A** The PPI for CXCR2. **B** The PPI for LMNB1. **C** TF-miRNA coregulatory network of CXCR2. **D** TF-miRNA coregulatory network of LMNB1

To determine the regulatory mechanism, the proteins closely related to CXCR2 and LMNB1 were predicted using the STRING database. The proteins showed close relationship with CXCR2 including CXCL1, CXCL2, CXCL3, CXCL5, CXCL6, CXCL8, PPBP, ARB1,CD74, and VASP (Fig. 9A). For LMNB1 protein network, LEMD3, LMNA, TMPO, EMD, LBR, BANF1, SUN1, SUN2, PPKCA, and CDK1 were predicted (Fig. 9B). The interaction between TFs and hub genes were analyzed, the result showed four TF-genes (CEBP1, NFKB1, RELA, USF2) regulated the expression of CXCR2. Meanwhile, nine TF-genes play roles in TF-LMNB1 regulatory network. In order to comprehensively understand the connection that contributes to controll the hub genes expression, microRNA-gene network analyses were also performed. The TF-miRNA coregulatory network was showed by Cytoscape tool (Fig. 9C,D). 23 and 157 miRNAs were predicted to be involved in the expression regulation of hub genes. The information of edges was listed in supplementary Table S4.

## Discussion

Polycystic ovary syndrome is a complex and intractable endocrinopathy, which exerts adverse influence on both reproduction and metabolism for women of childbearing age [10]. Cellular senescence is a condition of standing cell cycle arrest, which contribute great importance to aging and aging-associated diseases. Studies revealed that PCOS and ovarian aging share multiple common mechanisms in terms of pathogenesis such as oxidative stress, cellular metabolic process, mTOR pathway, and so on [11, 12]. Nonetheless, the underlying mechanism that promotes cell senescence and PCOS pathophysiology remain largely unknown. In this study, we aimed to reveal the intrinsic connection between PCOS and cell senescence, which may reveal the potential role of PCOS in female ovarian aging.

Recently, dozens of studies have been published to highlight the role of ARGs in various aging-related diseases. For instance, research by Xiao et al. probed into the potential role of aging-related genes in glioma [13]. Also, an aging-related signature genes for the prognosis of lung cancer was established [14]. Except for carcinomas, the role of ARGs in non-tumor conditions gained wide concern, such as asthma, pulmonary fibrosis, and so on [15, 16]. However, bioinformatic analysis focusing on ARGs in PCOS remains to be performed, and signature ARGs involved in PCOS pathogenesis are not determined.

In this study, we found the ARDEGs by intersecting DEGs and 1230 aging and senescence-associated genes. In addition, four hub ARDEGs (ANXA3, CXCR2, IQGAP2, and LMNB1) were identified by WGCNA and SVM-RFE algorithm, and the expression and diagnostic value was also validated using internal and external datasets.

The enrichment analysis of biological function for ARDEGs was performed. For GO analysis, it mainly focuses on immune-associated function, such as leukocyte cell migration, cytokine − mediated signaling pathway, and so. Similarly, GSEA method showed that those genes were mainly enriched in immune effector process, leukocyte mediated immunity, and cell adhesion. The function of the immune system declines during aging, actually, aging was also deemed as a chronic low-grade inflammatory state [17]. In the future, the molecular mechanism of immune in aging process needs further exploration.

The KEGG pathway investigation for ARDEGs indicated that those ARDEGs were primarily involved in NF-κB, PI3K-Akt, and JAK/STAT signal pathways. NF-κB pathway was revealed to be involved in the process of skin aging [18]. In osteoarthritis, a common aging-related degenerative joint disease, NF-κB signaling was also activated [19]. Previous research had linked the PI3K-Akt signal pathway to age-related diseases. Specially, it was found that the PI3K-Akt-mTOR signal pathway is active during hippocampus aging [20]. Furthermore, study showed that the drugs adjusting PI3K-Akt signal pathway played a role in anti-aging [21]. Above all, the result of functional enrichment showed that the ARDEGs in PCOS had close relationship within aging process, the role of those ARDEGs in ovarian aging deserves our attention and further studying.

To find the most important hub genes among the 73 ARDEGs, we performed WGCNA and SVM-RFE analysis to find 4 signature genes. Then the expression and diagnostic activity were assessed using both training and testing datasets. Besides, relative expression quantification was also performed in senescent GCs and GCs from PCOS patients by qRT-PCR. CXCR2 and LMNB1 were confirmed the excellent importance in PCOS and aging. Notably, CXCR2 showed significant up-regulation in both aged GCs and GCs of PCOS group, which indicated its potential role in promoting GCs senescence. However, expression trend of LMNB1 in PCOS GCs (up-regulated) is contrary to that in aGCs (down-regulated). This hinted us that PCOS might be a protective factor for ovarian aging and the underlying mechanism requires further exploration in the future.

We reviewed the role of CXCR2 and LMNB1 in PCOS and aging. Firstly, we found that CXCR2 showed great importance in senescence and tumor. Senescent cancer cells promoted CXCR2 expression and mediated the resistance against therapy, meanwhile, inhibition of CXCR2 was found to promote cancer cell senescence process, then improved the survival period of patients with Head-and-Neck and lung carcinoma [22, 23]. Also, the studies indicated that the gene was important in other cell aging-associated conditions, like depression, Alzheimer’s disease (AD), pathological healing, and atrophic Gastritis [24–27]. Recently, researchers found that inhibition of CXCL5-CXCR2 signal pathway in aged mouse embryos could improve the implantation rate, increasing the pregnancy rates and the delivery of normal pups [28]. In terms of mechanism of CXCR2, senescence-induced alterations in genes expression of CXCR2 may interacted with C-X-C motif chemokine ligand (CXCL) and promoted the proliferation and migration of pancreatic stellate cells [29]. Blockade of CXCR2 through toll-like receptor (TLR)2, and TLR5 signaling inhibited the secretion of interukin-8 (IL-8) or growth-regulated oncogene-alpha (GROa) by senescent mesenchymal stem cells [30]. Additionally, CXCR2 and p53 formed a positive feedback loop to constantly enhance cellular senescence in helicobacter pylori-induced atrophic gastritis [25]. CXCR2 knockout monocytes infused mice differentiated in TNF-α-releasing macrophages, leading to cell senescence [31]. IL-8/CXCR2 signaling pathway were found to accelerate senescence of placenta-derived mesenchymal stem cells [32].

Functional effect of nuclear LMNB1 was also studied, according to the cellular localization and function, the gene are crucially involved in nuclear processes like chromatin organization. This nuclear lamina degradation upon aberrant cellular stress induced cellular senescence, also, the loss of methylated LMNB1 played roles in aged human cells [33]. Besides, LMNB1 knockdown experiment revealed the implication in important cellular processes driving ER stress leading to cell senescence in malignant melanoma [34].

Besides, pathway analysis was performed for CXCR2 and LMNB1, and the result indicated an intrisic connection between CXCR Chemokine Receptors and Aging process (Table S5). Specifically, Aging is associated with changes in the immune system, a process known as immunosenescence. These changes can affect the function and expression of various immune receptors, including chemokine receptors like CXCR2. Also, these genes regulate the chemotaxis of neutrophils and leukocytes to some degree as identified by the PPI and pathway analysis above. The expression and function of chemokine receptors may be altered with age, and this can influence immune cell trafficking and responses to inflammation. Inflammation is directly associated with PCOS, which might help you tie things together.

Since the ARDEGs analysis of GO and GSEA were mostly enriched in immune pathway, which indicated the immune cells and immune dysfunction have relation to PCOS and cellular senescence. In this study, no different immune infiltration of immunocytes were found between groups, notably, correlation analysis revealed a significant connection between hub genes and immunocytes. Spearman analysis revealed that CXCR2 and LMNB1 were negatively correlated with macrophage M1, and CXCR2 was positively related to neutrophils. Furthermore, LMNB1 was negatively correlated with macrophage M2, B naïve, and plasma cells. PCOS is associated with increased neutrophils, which corelated positively with two hub biomarkers. Besides, PCOS is characterized by a shift from an anti-inflammatory M2 to a proinflammatory M1 state, yet, our finding of opposite connection of CXCR2 and LMNB1 with macrophage M1 indicated that the signature genes and the cell in PCOS need to be further clarified [35]. Besides, the chronic inflammation caused by imbalanced immunocytes may contribute to tissue fibrosis, one of the main mechanistic pillars of aging, and ultimately results in ovarian aging. As far as we know, CXCR2 and LMNB1 have not been studied in PCOS before; however, we speculated their latent roles in the immune process of PCOS. In the future, further research is required to reveal the intricate relationship between the hub genes and immune infiltration in both PCOS and aging.

To find the regulatory molecules among the 2 signature genes, we constructed a TF-miRNA-mRNA coregulatory network. miRNAs take part in cell senescence pathophysiology and have influence on the disease progression. According to the analysis result, the two hub genes were both regulated by hsa-mir-194-5p, hsa-mir-27a-3p, and hsa-mir-429. By examining the interaction between hub genes, TFs and miRNAs, we discovered that several miRNAs are implicated in PCOS (such as hsa-mir-29a-5p, hsa-mir-320, hsa-mir-429, hsa-mir-103a-3p, hsa-mir-192-5p, hsa-mir-145-5p, hsa-mir-218-5p, hsa-mir-1224-5p, hsa-mir-27a-3p) [36–42] and aging (such as hsa-mir-194-5p, hsa-mir-26b-5p, has-let-7e-5p, hsa-mir-181a-5p, hsa-mir-30a-3p, hsa-mir-200b-3p, hsa-mir-200c-3p, hsa-mir-30a-5p, hsa-mir-130a-3p, hsa-mir-506-3p, hsa-mir-93-5p, hsa-mir-148b-3p, hsa-mir-15b-5p, hsa-mir-17-3p) [43–53]. Some miRNAs have dual roles in cellular senescence and PCOS including hsa-mir-22-3p, hsa-mir-335-5p, 126-3p, 21-5p, 23a-3p, 124-3p, 122-5p, 34a-5p, 125a-5p, 125b-5p, 155-5p, 760, 199a-5p, and 424-5p [38, 45, 46, 54–72].

Several limitations of the study require to be noted. Firstly, the analysis is mainly based on the public datasets, which contained relatively small sample size. Thus, the results may be biased, and subsequent research with expand samples is required for obtaining more reliable conclusions. Secondly, screening of hub gene is mainly based on WGCNA and computational algorithm, and the results are hypothetical, in vitro and in vivo experiments are necessary to confirm the hub genes in PCOS and aging. Therefore, we can build on the existing research to provide a theoretical framework and foundation for further exploring the relation between PCOS and cellular senescence through gain or loss of function on biological models.

## Conclusions

Altogether, our research determined two potential senescence-associated hub genes (CXCR2, and LMNB1) with good diagnostic properties in PCOS using bioinformatic analysis and machine learning methods. Furthermore, we constructed a TF-miRNA coregulatory pathway for the genes. The characteristic genes were remarkably linked with immunological functions, besides, CXCR2 and LMNB1 per se and their regulatory miRNAs showed close connection with multiple cellular senescence pathways. These genes may affect the occurrence and prognosis of PCOS by regulating senescence. The above findings deepen our understanding of the unique relationship between cellular senescence and PCOS, and indicate that in-depth study of PCOS may provide insight into the process of granulosa cell senescence. Based on the results from bioinformatic analysis, we can provide a theoretical framework and foundation for further exploring the relation between PCOS and cellular senescence through gain or loss of function on biological models. Further research is needed, which may lay the theoretical foundation for future studies in ovarian aging and improve reproductive lifespan for women.

## Methods

### Data collection and processing

Microarray data, including four transcription profiles [GSE34526, GSE102293, GSE114419, GSE106724] were downloaded from the NCBI GEO database. Inclusion criteria was as follows: (1) 2003 Rotterdam criteria was as the basis for PCOS diagnosis; (2) mural GCs were isolated from follicular fluid aspirates; (3) unaffected women were selected as a control group (notably, the control part included women of childbearing age with an infertile male partner, tubal obstruction, or unexplained infertility seeking for IVF); (4) quantitative real-time PCR (qRT-PCR) was performed for experimental validation. GSE34526, GSE102293, GSE114419 datasets were used as the training dataset and GSE106724 was used to validate the hub genes. The ComBat method was used to normalize the data. Raw data was processed by R statistical software (version 4.2.1) and “Bioconductor” tools. Genes without being simultaneously included in there datasets were excluded. The “sva” R package was applied to remove the batch effect.

### Aging and senescence-associated genes

Aging and senescence-associated genes were searched from the Human Aging Genomic Resources2 (https://genomics.senescence.info/genes/index.html) and MSigDB gene sets3 [including datasets M16568, M24705, M14683, M27188, M13158, M14701, M11850, M40058, M39619] (https://www.gsea-msigdb.org/gsea/msigdb). Besides, a new senescence-related gene set [SenMayo] discovered by Mayo clinic was also included in this study [73].

### Differential expression genes analysis

The “limma” package was used for analyzing differentially expressed genes in granulosa cells between control group and women with PCOS. And *P*-value < 0.05 with |Fold Change|> 1.5 were as the threshold for selecting DEGs. The significantly up-regulated and down-regulated genes were visualized through Heatmap and volcano plot using “Pheatmap” and “ggplot2” packages, respectively.

### Identification and function enrichment analysis of ARDEGs

The Venn online tool (https://bioinfogp.cnb.csic.es/tools/venny/index.html) was applied for screening intersections between differential expression genes and cellular senescence-associated genes, namely, ARDEGs, which was visualized with heatmap plot using the “Pheatmap” package.

We used the “ClusterProfiler” R package to enrich the biological function of ARDEGs. GO analysis identified the biological phenomena of DEGs, which primarily consists of biological processes (BP), cellular components (CC), and molecular functions (MF). Moreover, pathway enrichment was done using KEGG. GSEA on cellular and senescence-associated DEGs was also performed using the “c5.all.v7.0.entrez.gmt” gene set, which was downloaded from the MSigDB database.

### Construction of the co-expression network and hub ARDEGs identification

WGCNA is an algorithm to cluster genes into different modules and uncover the relationships between modules and disease traits [74]. To identify the key modules involved in the pathogenesis of PCOS, we used “WGCNA” package to perform the WGCNA. The analysis included following four steps: 1) Genes with top 5000 variance were selected to constructed the co-expressed network and gene module; the soft threshold power was set to the scale-free topology criterion (signed R^2^ = 0.85). 2) To assess network connectivity, acquired weighted adjacency matrix was transformed into a topological overlap matrix. 3) The matrix was then used to generate a clustering tree using the average-linkage hierarchal clustering method. 4) The genes were classified according to their expression patterns; in this way, the genes were separated into different modules.

SVM-RFE algorithm was also performed to screen out the aging-related crucial genes in PCOS patients. SVM is a machine learning method and has been widely used for classification and regression [75]. The SVM-RFE requires multiple classifiers to obtain the total ranking criterion score, which is used as the basis for feature rejection, so as to avoid over-fitting and search for the best features.

In this study, genes overlapped in DEGs, genes from WGCNA key modules, and ARGs were intersected with important genes identified by SVM-RFE algorithm to identify hub ARDEGs.

### Expression validation and predicted performance of feature genes

To verify the above bioinformatics analyses, we draw the ROC curve to further evaluate the diagnostic capability of candidate biomarkers using “pROC” package in R software. *P* < 0.05 indicated the difference is statistically significant. Furthermore, internal and external datasets were also used for validating the expression of potential hub genes.

### Review the role of hub genes in PCOS and aging

The basic and functional information for aging-related PCOS signature genes was achieved obtained from the BioGPS website (http:// biogps.org), the Human Protein Atlas (https://www.proteinatlas.org/), and GeneCards (https://www.genecards.org/). Besides, the Pubmed database was systematically searched with the keyword “CXCR2”, “LMNB1”, “PCOS”, “Polycystic Ovary Syndrome”, “aging”, and “senescence”. Articles that studied the relationship between hub genes and PCOS/aging were reviewed.

### Immune infiltration analysis and its correlation with senescence-associated hub genes

Based on the microarray data, CIBERSORT research was performed to predict the differential abundance of 22 leukocyte subtypes between PCOS and control groups [76]. Bar plot of fractions and boxplot were draw by using the “ggplot2” package, respectively. Further, we used Spearman’s method for the correlation analysis between senescence-associated signature genes expression and the content of immune cells, *p* < 0.05 indicated significant difference.

### Regulatory network analysis of CXCR2 and LMNB1

microRNA and TF usually take part in the gene transcription and post-transcription regulation. To explore the regulatory network and further identify the intrinsic associations of hub genes with PCOS and aging, the protein–protein interaction (PPI) network, TF, and microRNA that interacted with CXCR2 and LMNB1 were analyzed. The STRING online database (https://string-db.org/) was applicated to study hub gene and protein interactions. The Cistrome DB database (http://cistrome.org/db/#/) was searched for finding TFs of hub genes. MicroRNAs those targeting genes were also screened from miRNANet database (https://www.mirnet.ca/miRNet/home.xhtml). Then, we incorporated the TF-miRNA coregulated interactions. Cytoscape software (version 3.7.2) was used to visualize the PPI network and interactions between TFs, microRNAs, and signature genes.

### Cell line culture and etoposide treatment

KGN, the human granulosa-like tumor cell line, was purchased from the Shanghai Fuheng Biotechnology. KGN cell line was cultured in DMEM/F12 medium containing 10% fetal bovine serum, 100 U/mL of penicillin and 100 μg/mL of streptomycin. Those cells were grown in 95% humidified air and 5% CO2 at 37°C. Cells were exposed to 5 μM etoposide for 48 h (in complete medium) to trigger cellular senescence. And cells were allowed a recovery period of 24 h prior to the following experiments.

### Senescence-associated β-galactosidase staining (SA-β-gal assay)

SA-β-gal assay was applied to identify KGN senescence. KGN cells were seeded on a 24-well plate at a density of 2 ∗ 10^4^ cells/well. After fixation, cells were stained with SA-β-gal staining solution overnight at 37°C and in CO2-free environment (C0602, Beyotime Biotechnology). Cells were imaged and photographed under a light microscope, and then we manually counted 3 fields of SA-β-gal-positive cells to determine the percentage.

### Patients and sample acquisition

Between September 2021 and October 2022, we recruited 30 women who underwent in vitro fertilization and embryo transfer (IVF-ET) at the Reproductive Center of Qilu Hospital, Shandong University. 15 patients were diagnosed as PCOS based on the Rotterdam criteria, and 15 control patients were infertile due to either fallopian tube obstruction or husband’s subfertility.

After the ovarian stimulation, the follicular fluid was extracted under ultrasound echo guidance and immediately centrifuged for 10 min at 1000 g. We suspended the precipitates with lymphocyte separation medium (P8610, Solarbio), and extracted the intermediate layer cells (human Granulosa Cell, hGCs) after the centrifugation. hGCs were then stored at − 80 °C until RNA extraction.

### Quantitative real‑time PCR (RT‑qPCR)

RNA extraction was performed from KGN cells and hGCs using RNA-easy Isolation Reagent (R701, Vazyme), and then total RNA was reverse-transcribed into cDNA with an RT Kit (11142ES60, Yesen). Each PCR system (10 μL) contained 5μL SYBR Green (11184ES03, Yesen), 30 ng cDNA and 300 nmol/L specific primers. We listed the primer sequence of each gene in Supplementary Table S1. And the PCR program consisted of 2 min at 95°C, followed by 40 cycles of 95°C for 10 s and 60°C for 30 s. We calculated the gene expression using the 2-ΔΔCT method and β-Actin was as the internal control.

### Statistical analysis

Expression data for granulosa cells obtained by RT-qPCR was analysed with t-test using the GraphPad Prism version 8.0.2 software. Differences were considered statistically significant when the *P*-value was < 0.05.

### Supplementary Information

**Additional file 1:** **Table S1.** The primer sequences used in qRT-PCR.

**Additional file 2:** **Table S2.** List of aging and senescence-associated genes.

**Additional file 3:** **Table S3.** Gene lists of the WGCNA key modules and SVM-RFE algorithm.

**Additional file 4:** **Table S4.** The edges information for TF-miRNA-mRNA regulatory network.

**Additional file 5:** **Table S5.** The pathway information for CXCR2 and LMNB1.

## Data Availability

Microarray data in this work are available in the GEO online database (http://www.ncbi.nlm.nih.gov/geo).

## Abbreviations

PCOS: Polycystic ovary syndrome
DEGs: Differentially expressed genes
ARGs: Aging-related genes
ARDEGs: Aging-related differential expression genes
WGCNA: Weighted gene co-expression network analysis
SVM-RFE: Support vector machine recursive feature elimination
AUC: Area under the curve
GEO: Gene Expression Omnibus
GO: Gene ontology
BP: Biological processes
CC: Cellular components
MF: Molecular functions
KEGG: Kyoto encyclopedia of genes and genomes
GSEA: Gene set enrichment analysis
PPI: Protein–protein interaction
TF: Transcriptional factor
miRNA: MicroRNA
GCs: Granulosa cells
aGCs: Aged granulosa cells
yGCs: Young granulosa cells
SA-β-gal assay: Senescence-associated β-galactosidase staining
ETO: Etoposide

## References

[1] Xia Q, Wang W, Liu Z, Xiao J, Qiao C, Zhao Y (2023). New insights into mechanisms of berberine in alleviating reproductive disorders of polycystic ovary syndrome: anti-inflammatory properties. Eur J Pharmacol.

[2] Yao X, Wang X (2022). Bioinformatics searching of diagnostic markers and immune infiltration in polycystic ovary syndrome. Front Genet.

[3] Roy AL, Sierra F, Howcroft K, Singer DS, Sharpless N, Hodes RJ, Wilder EL (2020). A blueprint for characterizing senescence. Cell.

[4] Liu C, Peng J, Matzuk MM, Yao HHC (2015). Lineage specification of ovarian theca cells requires multicellular interactions via oocyte and granulosa cells. Nat Commun.

[5] Jozkowiak M, Piotrowska-Kempisty H, Kobylarek D, Gorska N, Mozdziak P, Kempisty B (2022). Endocrine disrupting chemicals in polycystic ovary syndrome: the relevant role of the theca and granulosa cells in the pathogenesis of the ovarian dysfunction. Cells.

[6] Rb G, Lj R, Dt A. Oocyte-Somatic Cell Interactions during Follicle Development in Mammals. Animal reproduction science. 2004; 82–83. 10.1016/j.anireprosci.2004.05.017 15271471

[7] de Magalhães JP, Passos JF (2018). Stress, cell senescence and organismal ageing. Mech Ageing Dev.

[8] Wu T, Gao YY, Tang XN, Li Y, Dai J, Zhou S (2022). Construction of a competing endogenous RNA network to identify drug targets against polycystic ovary syndrome. Hum Reprod.

[9] Cozzolino M, Herraiz S, Titus S, Roberts L, Romeu M, Peinado I (2022). Transcriptomic landscape of granulosa cells and peripheral blood mononuclear cells in women with PCOS compared to young poor responders and women with normal response. Hum Reprod.

[10] Abolhassani N, Winterfeld U, Kaplan YC, Jaques C, Minder WB, Del GC (2023). Major malformations risk following early pregnancy exposure to metformin: a systematic review and meta-analysis. BMJ Open Diabetes Res Care.

[11] Ho CH, Chang CM, Li HY, Shen HY, Lieu FK, Wang PSG (2020). Dysregulated immunological and metabolic functions discovered by a polygenic integrative analysis for PCOS. Reprod Biomed Online.

[12] Dong L, Teh DBL, Kennedy BK, Huang Z (2023). Unraveling female reproductive senescence to enhance healthy longevity. Cell Res.

[13] Xiao G, Zhang X, Zhang X, Chen Y, Xia Z, Cao H (2021). Aging-related genes are potential prognostic biomarkers for patients with gliomas. Aging (Albany NY).

[14] Xu Q, Chen Y (2021). An aging-related gene signature-based model for risk stratification and prognosis prediction in lung adenocarcinoma. Front Cell Dev Biol.

[15] Yang Y, Yuan L, Yang M, Du X, Qin L, Wang L (2021). Aberrant methylation of aging-related genes in asthma. Front Mol Biosci.

[16] He J, Li X (2022). Identification and validation of aging-related genes in idiopathic pulmonary fibrosis. Front Genet.

[17] Cisneros B, García-Aguirre I, Unzueta J, Arrieta-Cruz I, González-Morales O, Domínguez-Larrieta JM (2022). Immune system modulation in aging: molecular mechanisms and therapeutic targets. Front Immunol.

[18] Trentini M, Zanolla I, Zanotti F, Tiengo E, Licastro D, Dal Monego S (2022). Apple derived exosomes improve collagen type i production and decrease MMPs during aging of the skin through downregulation of the NF-ΚB pathway as mode of action. Cells.

[19] Lu R, He Z, Zhang W, Wang Y, Cheng P, Lv Z (2022). Oroxin B alleviates osteoarthritis through anti-inflammation and inhibition of PI3K/AKT/MTOR signaling pathway and enhancement of autophagy. Front Endocrinol (Lausanne).

[20] Meng S, Xia W, Pan M, Jia Y, He Z, Ge W (2020). Proteomics profiling and pathway analysis of hippocampal aging in rhesus monkeys. BMC Neurosci.

[21] Gong P, Wang D, Cui D, Yang Q, Wang P, Yang W (2021). Anti-aging function and molecular mechanism of radix astragali and radix astragali preparata via network pharmacology and PI3K/Akt signaling pathway. Phytomedicine.

[22] Schoetz U, Klein D, Hess J, Shnayien S, Spoerl S, Orth M (2021). Early senescence and production of senescence-associated cytokines are major determinants of radioresistance in head-and-neck squamous cell carcinoma. Cell Death Dis.

[23] Cheng Y, Mo F, Li Q, Han X, Shi H, Chen S (2021). Targeting CXCR2 inhibits the progression of lung cancer and promotes therapeutic effect of cisplatin. Mol Cancer.

[24] Zhu YJ, Fan JJ, Wu FY, Zhang M, Song AQ, Li Y (2022). Aging promotes chronic stress-induced depressive-like behavior by activating NLRP1 inflammasome-driven inflammatory signaling in mice. Inflammation.

[25] Cai Q, Shi P, Yuan Y, Peng J, Ou X, Zhou W (2021). Inflammation-associated senescence promotes helicobacter pylori-induced atrophic gastritis. Cell Mol Gastroenterol Hepatol.

[26] Patel D, Zhang X, Farrell JJ, Lunetta KL, Farrer LA (2021). Set-based rare variant expression quantitative trait loci in blood and brain from alzheimer disease study participants. Genes (Basel).

[27] Wilkinson HN, Hardman MJ (2021). Wound senescence: a functional link between diabetes and ageing?. Exp Dermatol.

[28] Kawagoe Y, Kawashima I, Sato Y, Okamoto N, Matsubara K, Kawamura K (2020). CXCL5-CXCR2 signaling is a senescence-associated secretory phenotype in preimplantation embryos. Aging Cell.

[29] Takikawa T, Hamada S, Matsumoto R, Tanaka Y, Kataoka F, Sasaki A, Masamune A (2022). Senescent human pancreatic stellate cells secrete CXCR2 agonist CXCLs to promote proliferation and migration of human pancreatic cancer AsPC-1 and MIAPaCa-2 cell lines. Int J Mol Sci.

[30] Kwon JH, Kim M, Um S, Lee HJ, Bae YK, Choi SJ (2021). Senescence-associated secretory phenotype suppression mediated by small-sized mesenchymal stem cells delays cellular senescence through TLR2 and TLR5 signaling. Cells.

[31] Di Mitri D, Mirenda M, Vasilevska J, Calcinotto A, Delaleu N, Revandkar A (2019). Re-education of tumor-associated macrophages by CXCR2 blockade drives senescence and tumor inhibition in advanced prostate cancer. Cell Rep.

[32] Li JJ, Ma FX, Wang YW, Chen F, Lu SH, Chi Y (2017). Knockdown of IL-8 provoked premature senescence of placenta-derived mesenchymal stem cells. Stem Cells Dev.

[33] Rao RA, Ketkar AA, Kedia N, Krishnamoorthy VK, Lakshmanan V, Kumar P (2019). KMT1 family methyltransferases regulate heterochromatin-nuclear periphery tethering via histone and non-histone protein methylation. EMBO Rep.

[34] Lämmerhirt L, Kappelmann-Fenzl M, Fischer S, Pommer M, Zimmermann T, Kluge V (2022). K. Knockdown of Lamin B1 and the corresponding lamin B receptor leads to changes in heterochromatin state and senescence induction in malignant melanoma. Cells.

[35] Luan YY, Zhang L, Peng YQ, Li YY, Liu RX, Yin CH (2022). Immune regulation in polycystic ovary syndrome. Clin Chim Acta.

[36] Deswal R, Dang AS (2020). Dissecting the role of Micro-RNAs as a diagnostic marker for polycystic ovary syndrome: a systematic review and meta-analysis. Fertil Steril.

[37] Mao Z, Li T, Zhao H, Qin Y, Wang X, Kang Y (2021). Identification of epigenetic interactions between MicroRNA and DNA methylation associated with polycystic ovarian syndrome. J Hum Genet.

[38] De Nardo MG, Baracat EC, Soares JM, Carvalho KC, Maciel GAR (2022). Evaluation of circulating MicroRNA profiles in Brazilian women with polycystic ovary syndrome: a preliminary study. PLoS ONE.

[39] Zhang F, Li SP, Zhang T, Yu B, Zhang J, Ding HG (2021). High Throughput MicroRNAs sequencing profile of serum exosomes in women with and without polycystic ovarian syndrome. PeerJ.

[40] Patil K, Joseph S, Shah J, Mukherjee S (2022). An Integrated in silico analysis highlighted angiogenesis regulating MiRNA-MRNA network in PCOS pathophysiology. J Assist Reprod Genet.

[41] Li Y, Yao N, Gao Y, Wang Y, Bai L, Xu J (2021). MiR-1224-5p attenuates polycystic ovary syndrome through inhibiting NOD-like receptor protein 3 inflammasome activation via targeting forkhead box O 1. Bioengineered.

[42] Wang M, Sun J, Xu B, Chrusciel M, Gao J, Bazert M (2018). Functional characterization of MicroRNA-27a-3p expression in human polycystic ovary syndrome. Endocrinology.

[43] Liebig JK, Kuphal S, Bosserhoff AK (2020). HuRdling Senescence: HuR Breaks BRAF-induced senescence in melanocytes and supports melanoma growth. Cancers (Basel).

[44] Liu L, Zhao C, Zhang H, Lu Y, Luo B, Yao Z (2022). Asporin regulated by MiR-26b-5p mediates chondrocyte senescence and exacerbates osteoarthritis progression via TGF-Β1/Smad2 pathway. Rheumatology (Oxford).

[45] Han J, Li W, Zhang J, Guan Y, Huang Y, Li X (2022). Mechanism of CircHIPK3-MiRNA-124-3p/MiRNA-148b-3p-mediated inflammatory responses and cell senescence in Candida albicans-induced septic acute kidney injury. Gerontology.

[46] Markopoulos GS, Roupakia E, Tokamani M, Vartholomatos G, Tzavaras T, Hatziapostolou M (2017). Senescence-associated MicroRNAs target cell cycle regulatory genes in normal human lung fibroblasts. Exp Gerontol.

[47] Terlecki-Zaniewicz L, Lämmermann I, Latreille J, Bobbili MR, Pils V, Schosserer M, Weinmüllner R (2018). Small extracellular vesicles and their MiRNA cargo are anti-apoptotic members of the senescence-associated secretory phenotype. Aging (Albany NY).

[48] Moimas S, Salton F, Kosmider B, Ring N, Volpe MC, Bahmed K (2019). MiR-200 family members reduce senescence and restore idiopathic pulmonary fibrosis type II alveolar epithelial cell transdifferentiation. ERJ Open Res.

[49] Chevalier FP, Rorteau J, Ferraro S, Martin LS, Gonzalez-Torres A, Berthier A (2022). MiR-30a-5p alters epidermal terminal differentiation during aging by regulating BNIP3L/NIX-dependent mitophagy. Cells.

[50] Jiang X, Ruan XL, Xue YX, Yang S, Shi M, Wang LN (2020). Metformin reduces the senescence of renal tubular epithelial cells in diabetic nephropathy via the MBNL1/MiR-130a-3p/STAT3 pathway. Oxid Med Cell Longev.

[51] Borchardt H, Kogel A, Kalwa H, Weirauch U, Aigner A (2022). Therapeutic MiR-506-3p replacement in pancreatic carcinoma leads to multiple effects including autophagy, apoptosis, senescence, and mitochondrial alterations in vitro and in vivo. Biomedicines.

[52] Choi JY, Shin HJ, Bae IH (2018). MiR-93-5p suppresses cellular senescence by directly targeting Bcl-w and P21. Biochem Biophys Res Commun.

[53] Du WW, Li X, Li T, Li H, Khorshidi A, Liu F (2015). The MicroRNA MiR-17-3p inhibits mouse cardiac fibroblast senescence by targeting par4. J Cell Sci.

[54] Yuan D, Luo J, Sun Y, Hao L, Zheng J, Yang Z (2021). PCOS follicular fluid derived exosomal MiR-424-5p induces granulosa cells senescence by targeting CDCA4 expression. Cell Signal.

[55] Butler AE, Ramachandran V, Sathyapalan T, David R, Gooderham NJ, Benurwar M (2020). MicroRNA expression in women with and without polycystic ovarian syndrome matched for body mass index. Front Endocrinol (Lausanne).

[56] Chou J, Kaller M, Jaeckel S, Rokavec M, Hermeking H (2022). AP4 suppresses DNA damage, chromosomal instability and senescence via inducing MDC1/Mediator of DNA damage checkpoint 1 and repressing MIR22HG/MiR-22-3p. Mol Cancer.

[57] Liu Y, Yang J, Yang X, Lai P, Mou Y, Deng J (2022). H2O2 down-regulates SIRT7’s protective role of endothelial premature dysfunction via MicroRNA-335-5p. Biosci Rep.

[58] Zhang S, Liu Y, Wang M, Ponikwicka-Tyszko D, Ma W, Krentowska A (2022). Role and mechanism of MiR-335-5p in the pathogenesis and treatment of polycystic ovary syndrome. Transl Res.

[59] Jiang X, Li J, Zhang B, Hu J, Ma J, Cui L (2021). differential expression profile of plasma exosomal MicroRNAs in women with polycystic ovary syndrome. Fertil Steril.

[60] Accardi G, Bono F, Cammarata G, Aiello A, Herrero MT, Alessandro R (2022). miR-126-3p and miR-21-5p as Hallmarks of bio-positive ageing; correlation analysis and machine learning prediction in young to ultra-centenarian sicilian population. Cells.

[61] Jo H-R, Jeong J-H (2022). MicroRNA-mediated downregulation of HMGB2 contributes to cellular senescence in microvascular endothelial cells. Cells.

[62] Chen L, Kong C (2022). LINC00173 regulates polycystic ovarian syndrome progression by promoting apoptosis and repressing proliferation in ovarian granulosa cells via the MicroRNA-124-3p (MiR-124-3p)/Jagged Canonical Notch Ligand 1 (JAG1) pathway. Bioengineered.

[63] Huo Y, Ji S, Yang H, Wu W, Yu L, Ren Y (2022). differential expression of MicroRNA in the serum of patients with polycystic ovary syndrome with insulin resistance. Ann Transl Med.

[64] Tian-Min Y, Suxia L, Shufang D, Dandan C, Long-Dan L, Shu BY, W.  (2022). Combined transcriptomic and metabolomic analysis of women with polycystic ovary syndrome. Dis Markers.

[65] Romero-Ruiz A, Pineda B, Ovelleiro D, Perdices-Lopez C, Torres E, Vazquez MJ (2021). Molecular diagnosis of polycystic ovary syndrome in obese and non-obese women by targeted plasma MiRNA profiling. Eur J Endocrinol.

[66] Zhang X, Xiao H, Zhang XEQ, Gong X, Li T, Han Y (2020). Decreased MicroRNA-125b-5p disrupts follicle steroidogenesis through targeting PAK3/ERK1/2 signalling in mouse preantral follicles. Metabolism.

[67] Cao J, Huo P, Cui K, Wei H, Cao J, Wang J (2022). Follicular fluid-derived exosomal MiR-143-3p/MiR-155-5p regulate follicular dysplasia by modulating glycolysis in granulosa cells in polycystic ovary syndrome. Cell Commun Signal.

[68] Nguyen DDN, Zain SM, Kamarulzaman MH, Low TY, Chilian WM, Pan Y (2021). Intracellular and exosomal MicroRNAome profiling of human vascular smooth muscle cells during replicative senescence. Am J Physiol Heart Circ Physiol.

[69] Lee Y, Bae YS (2022). Long non-coding RNA KCNQ1OT1 regulates protein kinase CK2 Via MiR-760 in senescence and calorie restriction. Int J Mol Sci.

[70] Deng L, Chen Q, Xie J, Wei W, Hui H (2020). CircPUM1 promotes polycystic ovary syndrome progression by sponging to MiR-760. Gene.

[71] Tao W, Hong Y, He H, Han Q, Mao M, Hu B (2021). MicroRNA-199a-5p aggravates angiotensin II-induced vascular smooth muscle cell senescence by targeting sirtuin-1 in abdominal aortic aneurysm. J Cell Mol Med.

[72] Shao S, Wang H, Shao W, Liu N (2020). MiR-199a-5p stimulates ovarian granulosa cell apoptosis in polycystic ovary syndrome. J Mol Endocrinol.

[73] Zhang Q, Li J, Weng L (2022). Identification and validation of aging-related genes in Alzheimer’s Disease. Front Neurosci.

[74] Langfelder P, Horvath S (2008). WGCNA: an R package for weighted correlation network analysis. BMC Bioinformatics.

[75] Sanz H, Valim C, Vegas E, Oller JM, Reverter F (2018). SVM-RFE: selection and visualization of the most relevant features through non-linear kernels. BMC Bioinformatics.

[76] Newman AM, Liu CL, Green MR, Gentles AJ, Feng W, Xu Y (2015). Robust enumeration of cell subsets from tissue expression profiles. Nat Methods.

