# Supplementary material for: Identification and validation of senescence-related genes in polycystic ovary syndrome
Source: J Ovarian Res. 2024 Jan 6;17:7. doi: 10.1186/s13048-023-01338-4 (PMC10770899; doi:10.1186/s13048-023-01338-4)
Supplement: Supplementary file 1 — Additional file 1: Table S1. The primer sequences used in qRT-PCR. [file 13048_2023_1338_MOESM1_ESM.docx]

| Gene symbol | Forward primer | Reverse primer |
| --- | --- | --- |
| β-Actin | GAAGAGCTACGAGCTGCCTGA | CAGACAGCACTGTGTTGGCG |
| CXCR2 | CCTGTCTTACTTTTCCGAAGGAC | TTGCTGTATTGTTGCCCATGT |
| LMNB1 | TGGAAGAATCAGAGGCGAGT | TGCAGCCCAAATTGTAACAG |

Supplementary Table 1.The primer sequences used in qRT-PCR.
